# Extracorporeal photopheresis in BK virus infection after kidney transplantation: current evidence and emerging clinical applications

**DOI:** 10.1093/ckj/sfag251

**Published:** 2026-10-29

**Authors:** Ángela González, Marc Xipell, Víctor J Escudero-Saiz, Carolt Arana, Michael Nicoli, Jordi Rovira, Paola Charry, Marta Bodro, Pedro Ventura-Aguiar, Miquel Lozano, Fritz Diekmann

**Affiliations:** Department of Nephrology and Renal Transplantation, Hospital Clinic, Barcelona, Spain; Laboratori Experimental de Nefrologia I Transplantament Renal (LENIT), Fundació de Recerca Clínic Barcelona—Institut d’Investigacions Biomèdiques August Pi i Sunyer (IDIBAPS), Barcelona, Spain; Redes de Investigación Cooperativa Orientadas a Resultados en Salud (RICORS), Madrid, Spain; Department of Medicine, Faculty of Medicine and Health Sciences, University of Barcelona, Barcelona, Spain; Department of Nephrology and Renal Transplantation, Hospital Clinic, Barcelona, Spain; Laboratori Experimental de Nefrologia I Transplantament Renal (LENIT), Fundació de Recerca Clínic Barcelona—Institut d’Investigacions Biomèdiques August Pi i Sunyer (IDIBAPS), Barcelona, Spain; Redes de Investigación Cooperativa Orientadas a Resultados en Salud (RICORS), Madrid, Spain; Department of Medicine, Faculty of Medicine and Health Sciences, University of Barcelona, Barcelona, Spain; Department of Nephrology and Renal Transplantation, Hospital Clinic, Barcelona, Spain; Laboratori Experimental de Nefrologia I Transplantament Renal (LENIT), Fundació de Recerca Clínic Barcelona—Institut d’Investigacions Biomèdiques August Pi i Sunyer (IDIBAPS), Barcelona, Spain; Redes de Investigación Cooperativa Orientadas a Resultados en Salud (RICORS), Madrid, Spain; Department of Medicine, Faculty of Medicine and Health Sciences, University of Barcelona, Barcelona, Spain; Department of Nephrology and Renal Transplantation, Hospital Clinic, Barcelona, Spain; Laboratori Experimental de Nefrologia I Transplantament Renal (LENIT), Fundació de Recerca Clínic Barcelona—Institut d’Investigacions Biomèdiques August Pi i Sunyer (IDIBAPS), Barcelona, Spain; Redes de Investigación Cooperativa Orientadas a Resultados en Salud (RICORS), Madrid, Spain; Department of Medicine, Faculty of Medicine and Health Sciences, University of Barcelona, Barcelona, Spain; Laboratori Experimental de Nefrologia I Transplantament Renal (LENIT), Fundació de Recerca Clínic Barcelona—Institut d’Investigacions Biomèdiques August Pi i Sunyer (IDIBAPS), Barcelona, Spain; Department of Medicine, Faculty of Medicine and Health Sciences, University of Barcelona, Barcelona, Spain; Laboratori Experimental de Nefrologia I Transplantament Renal (LENIT), Fundació de Recerca Clínic Barcelona—Institut d’Investigacions Biomèdiques August Pi i Sunyer (IDIBAPS), Barcelona, Spain; Redes de Investigación Cooperativa Orientadas a Resultados en Salud (RICORS), Madrid, Spain; Department of Medicine, Faculty of Medicine and Health Sciences, University of Barcelona, Barcelona, Spain; Apheresis Unit, Department of Hemotherapy and Hemostasis, Hospital Clínic Barcelona, Catalonia, Spain; Department of Medicine, Faculty of Medicine and Health Sciences, University of Barcelona, Barcelona, Spain; Department of Infectious Diseases, Hospital Clínic Barcelona, Catalonia, Spain; Department of Medicine, Faculty of Medicine and Health Sciences, University of Barcelona, Barcelona, Spain; Department of Nephrology and Renal Transplantation, Hospital Clinic, Barcelona, Spain; Laboratori Experimental de Nefrologia I Transplantament Renal (LENIT), Fundació de Recerca Clínic Barcelona—Institut d’Investigacions Biomèdiques August Pi i Sunyer (IDIBAPS), Barcelona, Spain; Redes de Investigación Cooperativa Orientadas a Resultados en Salud (RICORS), Madrid, Spain; Department of Medicine, Faculty of Medicine and Health Sciences, University of Barcelona, Barcelona, Spain; Apheresis Unit, Department of Hemotherapy and Hemostasis, Hospital Clínic Barcelona, Catalonia, Spain; Department of Medicine, Faculty of Medicine and Health Sciences, University of Barcelona, Barcelona, Spain; Department of Nephrology and Renal Transplantation, Hospital Clinic, Barcelona, Spain; Laboratori Experimental de Nefrologia I Transplantament Renal (LENIT), Fundació de Recerca Clínic Barcelona—Institut d’Investigacions Biomèdiques August Pi i Sunyer (IDIBAPS), Barcelona, Spain; Redes de Investigación Cooperativa Orientadas a Resultados en Salud (RICORS), Madrid, Spain; Department of Medicine, Faculty of Medicine and Health Sciences, University of Barcelona, Barcelona, Spain

**Keywords:** BK virus, extracorporeal photopheresis, immunogenicity, immunological tolerance, kidney transplantation

## Abstract

BK polyomavirus (BKPyV) infection is a major complication after kidney transplantation, with no approved antiviral therapy and management reliant on immunosuppression reduction, which increases rejection risk. This review outlines the biological rationale for extracorporeal photopheresis (ECP) in infectious settings and critically appraises the current evidence for its use in BKPyV replication and BKPyV-associated nephropathy (BKPyVAN). ECP is an immunomodulatory therapy that induces apoptosis of apheresis-isolated mononuclear cells via *ex vivo* exposure to a photoactivatable agent and UVA light; reinfusion promotes tolerogenic dendritic cells, regulatory T-cell expansion, and cytokine modulation. Its antiviral potential was first observed in cytomegalovirus, with ECP linked to reduced viral burden in heart, lung, and hematopoietic transplantation. Experience in BKPyV is more limited, restricted to small case series and reports, where ECP combined with immunosuppression reduction was associated with sustained reductions in viremia, reaching >99% in individual series, without increased rejection. These uncontrolled, confounded designs cannot isolate an ECP-specific antiviral effect, and the proposed antiviral mechanism remains hypothetical. ECP may be a promising adjunctive strategy in selected high-risk patients with persistent BKPyV viremia or BKPyVAN, but prospective, controlled studies are needed to confirm efficacy and define optimal use.

## INTRODUCTION

BK polyomavirus (BKPyV) is a ubiquitous human polyomavirus, with adult seropositivity rates of 80%–90% [1, 2]. Following primary infection, it establishes lifelong latency in the urinary tract, with viral replication tightly controlled by T cell-mediated immunity under immunocompetent conditions. In kidney transplant recipients, immunosuppression disrupts this control, facilitating viral reactivation that may manifest as viruria, viremia, and ultimately BK polyomavirus-associated nephropathy (BKPyVAN) [3,5], with the overall intensity of immunosuppression as the main determinant of progression [6–11].

Management is particularly challenging in highly sensitized patients, in whom reduction of immunosuppression to control viral replication may increase the risk of rejection [12, 13], which has in turn been associated with an increased risk of de novo donor-specific antibodies and poorer graft outcomes [14, 15]. Overall, BKPyVAN remains a major cause of allograft dysfunction and graft loss despite advances in virological monitoring and immunosuppressive management, largely due to the absence of approved antiviral therapies with demonstrated clinical efficacy [16, 17].

This therapeutic gap has prompted growing interest in immunomodulatory strategies capable of restoring antiviral cellular immunity without broadly compromising graft tolerance. Extracorporeal photopheresis (ECP) is an immunomodulatory therapy with an established role in graft-versus-host disease and cardiac allograft rejection [18–20]. Its antiviral potential was first recognized in cytomegalovirus infection across heart, lung, and hematopoietic transplantation, and has more recently been explored in BKPyV, with case reports and small series suggesting reductions in viremia without increased rejection risk [21–24]. The aim of this review is to summarize the immunomodulatory effects of ECP in infectious contexts and to critically evaluate current evidence supporting its role as an emerging therapeutic strategy in patients with BKPyV replication or BKPyVAN.

## BKPyV INFECTION IN KIDNEY TRANSPLANTATION: DIAGNOSIS AND RISK STRATIFICATION

BKPyV replication is common after transplantation, with viruria occurring in 30%–50% of recipients and viremia in 10%–20%, progressing to BKPyVAN in ~5% of cases [6, 7]. The main determinant of progression is the overall intensity of immunosuppression: higher risk has been consistently associated with lymphocyte-depleting induction therapy, treatment of rejection episodes with high-dose corticosteroids, increased calcineurin inhibitor exposure, and B cell-depleting agents such as rituximab [8, 9, 10], whereas mTOR inhibitor-based regimens have been suggested in some series to confer lower risk, albeit with inconsistent evidence [11].

The diagnosis of BKPyV infection after kidney transplantation relies on a combination of virological and histological assessment. Quantitative polymerase chain reaction (PCR) monitoring of BKPyV DNAemia represents the cornerstone of early detection, with plasma BKPyV DNA levels persisting above 1000 copies/ml (probable BKPyVAN) or exceeding 10 000 copies/ml (presumptive BKPyVAN) considered highly suggestive and prompting timely immunosuppression reduction in the appropriate clinical context.

Kidney biopsy remains the gold standard for definitive diagnosis of BKPyVAN. Histological features include viral intranuclear inclusions in tubular epithelial cells, tubular injury, interstitial inflammation, and varying degrees of fibrosis [25]. Immunohistochemical staining for the SV40 large T antigen confirms active intragraft viral replication.

The Banff classification for polyomavirus nephropathy integrates intrarenal viral burden, assessed by the polyomavirus load (pvl) score, and the degree of chronic damage, reflected by the interstitial fibrosis (ci) score. This classification provides prognostic stratification, with graft loss rates increasing from ~5%–15% in class 1 to around 30% in class 2 and up to 50% in class 3 during follow-up. Notably, patients with persistent fibrosis (ci ≥ 2) despite resolved viral replication (pvl = 0) may present long-term graft outcomes comparable to those observed in advanced histological classes [25].

Beyond viral load and histological assessment, BKPyV-specific T-cell immune monitoring has emerged as a complementary tool: IFN-γ ELISPOT assays correlate with risk of BKPyV reactivation and nephropathy, with pooled sensitivity and specificity exceeding 85% in a recent meta-analysis, and have been proposed to guide individualized immunosuppression adjustment alongside viral load [26, 27].

## CURRENT MANAGEMENT STRATEGIES AND UNMET NEEDS

To date, no approved antiviral therapy with demonstrated clinical efficacy exists for BKPyV infection. Consequently, management relies primarily on controlled reduction of immunosuppression. Expert consensus and current guidelines recommend a stepwise approach, typically starting with lowering calcineurin inhibitor exposure, followed—if viremia persists—by reduction or discontinuation of mycophenolate. In selected cases, conversion to mTOR inhibitors may be considered, given their potentially lower impact on antiviral cellular immunity, although supporting evidence remains inconsistent. The objective of this strategy is to restore antiviral cellular immunity while minimizing the risk of acute rejection. However, this balance is often difficult to achieve, particularly in patients at high immunological risk [12].

Several adjunctive therapies, including intravenous immunoglobulin, leflunomide, and cidofovir, have been explored, but evidence supporting their efficacy remains limited and heterogeneous. In this context, interest has grown in immunomodulatory approaches capable of enhancing antiviral immune responses without substantially increasing rejection risk [12]. Among these, ECP has emerged as a promising therapeutic option.

Virus-specific T-cell therapy represents the most advanced competing immunotherapeutic strategy currently in development, encompassing both off-the-shelf and personalized donor-derived products. Posoleucel, an off-the-shelf, allogeneic, multivirus-specific T-cell product targeting BKPyV among other opportunistic viruses, is the only such therapy evaluated in a randomized, double-blind, placebo-controlled phase 2 trial in kidney transplant recipients with BK viremia, which—though underpowered for clinical efficacy—found a greater reduction in viremia with posoleucel than placebo, associated with an increase in circulating BKPyV-specific T cells; the trial was generally well tolerated, though three rejection episodes occurred in posoleucel recipients, none adjudicated as treatment-related [28]. Donor- or third-party-derived BKPyV-specific T cells have also shown reductions in viral load and a favorable safety profile in a phase 2 study across hematopoietic and solid organ transplant recipients, without graft-versus-host disease or rejection attributable to the infused cells [29]. Unlike ECP, these approaches directly restore virus-specific cellular immunity rather than acting through broader immunomodulation, and represent the most rigorously evaluated alternatives to immunosuppression reduction to date.

## ECP: BIOLOGICAL RATIONALE AND MECHANISMS OF ACTION

ECP represents a mechanistically distinct alternative to these strategies, acting through broader immunomodulatory pathways rather than direct virus-specific immune restoration. Initially developed for the treatment of cutaneous T-cell lymphoma, whose use has since expanded to a broad range of immune-mediated disorders and solid organ transplantation [30–32]. The procedure involves mononuclear cell (MNC) collection by apheresis, *ex vivo* incubation with 8-methoxypsoralen, and subsequent exposure to ultraviolet A (UVA) radiation, after which the treated cells are reinfused into the patient [33]. The interaction between psoralen and UVA induces photochemical DNA damage, leading to apoptosis of the treated MNC fraction [34–36].

The immunological effects of ECP are complex and multifactorial and are often conceptualized within the framework of “transimmunization”, whereby apoptotic cells generated during the procedure are processed by the host immune system [18, 37]. It has additionally been proposed that during extracorporeal circulation, monocytes come into contact with the plastic surfaces of the apheresis kit, driving monocyte differentiation into dendritic cells [38]. These dendritic cells may acquire a tolerogenic phenotype after internalizing apoptotic cell-derived antigens, although this mechanism is debated since apoptosis predominantly occurs within 48 h after ECP [38 40]. Ultimately, this results in apoptosis of activated effector T lymphocytes, modulation of cytokine responses with increased anti-inflammatory mediators such as IL-10 and reduced pro-inflammatory cytokines including TNF-α and IL-6, along with expansion of regulatory T cells and modulation of costimulatory pathways [41–44] (Fig. 1).

**Figure 1:**
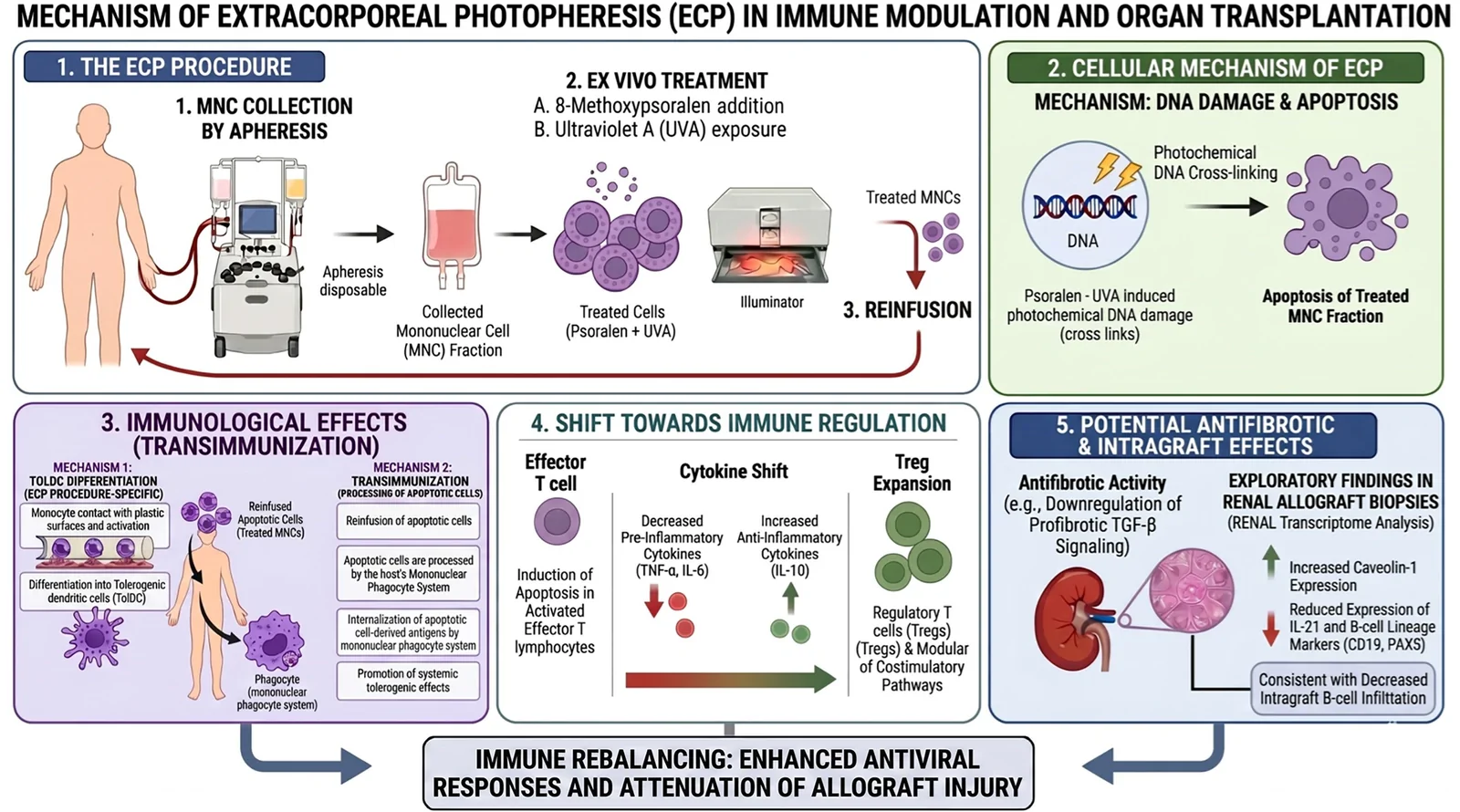
Mechanism of ECP in Immune Modulation and Organ Transplantation. (i) The ECP procedure. Patient MNCs are collected by apheresis, to which 8-methoxypsoralen is added and the exposed to UVA illumination, and subsequently reinfused. (ii) Cellular mechanism. The interaction between psoralen and UVA induces photochemical DNA cross-linking, leading to apoptosis of the treated MNC fraction. (iii) Immunological effects (transimmunization). Apoptotic cells generated during ECP are processed by the host immune system. In parallel, monocyte activation during extracorporeal circulation promotes differentiation into tolerogenic dendritic cells (tolDCs), which internalize apoptotic cell-derived antigens and favor immune tolerance. (iv) Shift toward immune regulation. ECP is associated with apoptosis of activated effector T lymphocytes, increased production of anti-inflammatory cytokines such as IL-10, decreased levels of pro-inflammatory mediators including TNF-α and IL-6, expansion of regulatory T cells (Tregs), and modulation of costimulatory pathways. (v) Potential antifibrotic and intragraft effects. ECP may downregulate profibrotic pathways such as TGF-β signaling and reduce intragraft B-cell infiltration, as suggested by decreased expression of CD19 and PAX5. Overall, these mechanisms may contribute to immune rebalancing and attenuation of chronic allograft injury.

A central, unresolved question is whether the tolerogenic mechanisms of ECP can translate into enhanced BKPyV-specific antiviral immunity. Viral clearance in BKPyV infection is primarily mediated by CD4 and CD8 T cells targeting viral antigens, and impaired clearance has been linked to dysfunctional, exhausted virus-specific T cells overexpressing inhibitory receptors such as PD-1, CTLA-4, and TIM-3; experimentally, blocking these receptors in combination can partially recover BKPyV-specific CD8 T cell function [45]. A marginal recovery of CD4 interferon-γ responses after immunosuppression reduction has been observed in BKPyVAN, though findings for CD8 responses have been inconsistent [46], and quantifiable CD8 responses against immunodominant epitopes correlate with resolution of viremia in non-nephropathy BKPyV viremia [47]. None of the available ECP studies in BKPyV infection have assessed these parameters; whether ECP reverses BKPyV-specific T cell exhaustion, restores antiviral memory responses, or acts exclusively through its established tolerogenic pathways without engaging virus-specific immunity remains unknown, and represents the central mechanistic gap underlying its proposed antiviral rationale.

Beyond these immunomodulatory effects, ECP may also exert antifibrotic activity, potentially through downregulation of profibrotic pathways such as TGF-β signaling [48]. In exploratory transcriptomic analyses of renal allograft biopsies from ECP-treated patients, modulation of genes involved in inflammation and fibrosis has been observed, including increased expression of caveolin-1 and reduced expression of IL-21 and B-cell lineage markers such as CD19 and PAX5, findings consistent with a decrease in intragraft B-cell infiltration [49]. Although these observations are derived from limited cohorts, they support the concept that ECP may contribute not only to immune rebalancing but also, indirectly, to attenuation of chronic allograft injury driven by persistent inflammation [50].

## EVIDENCE SUPPORTING THE ROLE OF ECP IN TRANSPLANT-RELATED INFECTIONS

An increasing number of publications have reported reductions in the incidence of viral infections among transplant recipients receiving ECP (Table 1).

**Table 1:** Studies evaluating infections in patients treated with ECP across different transplant settings.

Organ	Publication and study design	Population and treatment regimen	Infectious outcome assessed	Infectious outcomes
Lung	Benazzo *et al.*, 2025, *Eur Respir J*; prospective randomized trial	62 lung Tx; 31 ECP + standard IS vs. 31 control; ECP added during follow-up	Overall and severe infections; CMV replication and disease	Overall infections 64 (ECP) vs. 123 (control), *P* = 0.001; severe infections requiring hospitalization 5 vs. 22, *P* = 0.002; CMV replication 9/31 vs. 10/31; CMV disease 1/31 vs 2/31
Lung	Jaksch *et al.*, 2012, *J Heart Lung Transplant*; prospective interventional study	1012 lung Tx; 194 with BOS; 51 ECP + standard therapy	CMV disease and CMV viremia	Lower incidence of CMV disease and viremia in the ECP group than in controls
Lung	Pecoraro *et al.*, 2017, *Transplant Proc*; retrospective case series	239 lung Tx; 54 with BOS; 15 ECP	Overall infection incidence	ECP was not associated with an increased incidence of infections during follow-up
Heart	Barr *et al.*, 1998, *N Engl J Med*; randomized clinical trial	De novo heart Tx; standard IS ± ECP	Overall infection incidence	ECP reduced graft rejection without increasing the incidence of infections
Heart	Gökler *et al.*, 2022, *Transpl Int*; pilot study	28 high-risk heart Tx; reduced-intensity IS without induction + ECP	Safety; incidence of early post-operative infection at 6 months	ECP was safe, with a low rate of rejection, and an effective strategy in patients at high risk of infection or malignancy
Heart	Barten *et al.*, 2023, *J Heart Lung Transplant*; multicenter study	105 heart Tx; reduced-intensity IS; real-world ECP after rejection	Real-world use of ECP and associated complications	Infections were the most common complication: 13 patients (12.4%), of whom 8 (7.6%) had a non-CMV and 5 (4.8%) a CMV infection
Hematopoietic cell transplantation (GVHD)	Schain *et al.*, 2023, *Transfus Apher Sci*; population-based observational study	2 708 allo-HCT; 183 ECP for chronic GVHD	Infection incidence before and after ECP	Infection incidence 79.2% before vs 59.1% after ECP, *P* < 0.001
Hematopoietic cell transplantation (GVHD)	Gavriilaki *et al.*, 2024, *Pharmaceuticals*; clinical review	GVHD patients on ECP	Infectious complications	ECP showed a favorable safety profile, without an increase in opportunistic infections
Kidney (EBV-PTLD)	Augusto *et al.*, 2021, *Transpl Int*; case series	3 kidney Tx; EBV-PTLD + acute rejection; ECP	EBV/PTLD reactivation or recurrence	Favorable clinical course, with no PTLD relapse during follow-up
Kidney	Faenko *et al.*, 2018, *Urologiia*; open-label randomized trial	60 kidney Tx; 30 ECP + standard IS vs. 30 control; 10–15 ECP over first 6 mo	Respiratory infections, asymptomatic bacteriuria, viremia, and clinically meaningful infections	Respiratory infections 4-fold, asymptomatic bacteriuria 2-fold, and viremia 1.5-fold lower with ECP; risk of clinically meaningful infection lower with ECP (IRR 0.39, 95% CI 0.28–0.54, *P* < 0.0001)

Abbreviations: BOS, bronchiolitis obliterans syndrome; CMV, cytomegalovirus; ECP, extracorporeal photopheresis; EBV, Epstein–Barr virus; GVHD, graft-versus-host disease; HCT, hematopoietic cell transplantation; IRR, incidence rate ratio; IS, immunosuppression; PTLD, post-transplant lymphoproliferative disorder; and Tx, transplantation.

In heart transplantation, ECP has been associated with a lower incidence of CMV disease. In a multicenter randomized study published in 1998, with infection assessed as a secondary endpoint, 60 patients were assigned to receive either 24 ECP sessions during the first 6 months after transplantation or standard therapy. The ECP group had fewer rejection episodes (0.91 ± 1.0 vs. 1.44 ± 1.0 per patient; *P* = 0.04) and lower incidence of CMV replication (*P* = 0.04) [51, 52]. Barten *et al*. demonstrated that ECP is effective both for the treatment of various forms of rejection and for rejection prophylaxis following transplantation, importantly allowing a reduction in immunosuppressive burden, including corticosteroids and tacrolimus [53]. Prophylactic ECP administered for 6 months after transplantation allowed for reduced-intensity immunosuppression in recipients at increased risk of severe infections or cancer recurrence, while maintaining effective prevention of allograft rejection [54].

In lung transplantation, at least four studies reported reduced CMV infection with ECP. In a prospective study of 1012 recipients (194 with bronchiolitis obliterans syndrome, 51 ECP-treated), the ECP group showed lower rates of CMV replication (*P* = 0.01) and invasive CMV disease (*P* = 0.001) [20, 55]. Among responders, graft survival after bronchiolitis obliterans syndrome was higher, suggesting a potential link with reduced fibrosis. More recently, a study published in 2025 including 62 patients (31 treated with ECP) reported lower infection rates in the ECP group (5 vs. 22 cases; *P* = 0.002) [55–57].

In hematopoietic cell transplantation, ECP for graft-versus-host disease has been associated with reduced infections and corticosteroid exposure [58]. In a Swedish cohort of 183 adult patients, infection incidence decreased from 79.2% to 59.1% at 9–12 months after ECP initiation (*P* < 0.001), over a mean exposure of 7.8 months (18 sessions) [59, 60].

In kidney transplantation, a series of three cases treated for post-transplant lymphoproliferative disease showed clearance of Epstein–Barr virus viremia with ECP [61]. In addition, a single-center randomized study with limited methodological reporting, including 60 kidney transplant recipients showed that patients receiving 10–15 ECP sessions over 6 months had lower rates of respiratory infections, asymptomatic bacteriuria, and viremia (4-fold, 2-fold, and 1.5-fold reductions, respectively). The incidence rate ratio for clinically significant infection was 0.39 (95% CI 0.28–0.54; *P* < 0.0001) compared with standard immunosuppression alone [62, 63]. Given the immunobiological differences between CMV and BKPyV, and since the underlying mechanisms remain incompletely understood, this evidence remains hypothesis-generating rather than established; nonetheless, these findings support the biological plausibility of an antiviral role for ECP.

## CLINICAL EXPERIENCE OF ECP IN BKPyV INFECTION

One of the earliest clinical experiences was a 4-case series published by our group in 2021, including two patients at high infectious risk following acute rejection and intensive immunosuppression, and two additional patients with viral infections due to CMV and BKPyV. In the latter, BKPyVAN developed after prior treatment for antibody-mediated rejection. Patients received a median of ~11 ECP sessions in combination with immunosuppression reduction. Following ECP initiation, BKPyV viral load decreased by 90% at 14 days and by 99.9% at 6 months, with viremia rebounding after ECP discontinuation in one patient. Although this single-case finding does not establish causality, it is the most suggestive signal of a treatment-specific effect among the available data [21].

In 2024, a French group reported a case of early BKPyV replication occurring within the first month after kidney transplantation in a recipient with prior lung transplantation. Management consisted of immunosuppression minimization, including mycophenolate withdrawal, combined with ECP. This strategy was associated with progressive viral load decline, complete negativization at 9 months, absence of nephropathy, and stable renal function, suggesting a potential role of ECP in preventing disease progression while mitigating rejection risk [22].

A subsequent case reported by an Italian group in 2025 described a high immunological risk recipient who developed donor-specific antibodies after immunosuppression reduction for BKPyV viremia (peak viral load 40 500 copies/ml) 4 years post-transplantation. ECP was initiated as a supportive immunomodulatory strategy to reduce the risk of antibody-mediated rejection, with a total of 32 sessions administered over 13 weeks [23].

The largest series to date, also from our group, included 13 kidney transplant recipients with biopsy-proven BKPyVAN refractory to conventional management. Notably, 54% of patients were at high immunological risk (cPRA > 50%). ECP was initiated a median of 21 weeks after BKPyVAN diagnosis, with patients receiving a median of 11 sessions. Following treatment initiation, a sustained and progressive reduction in viremia was observed, with decreases of 55%, 96%, 99.7%, and 99.9% at 3, 6, 12, and 18 months, respectively. Renal function declined slowly, with a median eGFR loss of −0.69 ml/min/1.73 m² per month. Despite ongoing immunosuppression reduction, only one patient developed chronic active antibody-mediated rejection during follow-up, with no major impact on graft function and no treatment-related adverse events. Graft loss due to BKPyVAN progression occurred in two patients (15%) [24]. These findings are summarized in Table 2. Taken together, clinical experience with ECP in BKPyV infection remains confined to these few cases and series, precluding firm conclusions regarding efficacy.

**Table 2:** Studies evaluating extracorporeal photopheresis in kidney transplant recipients with BK virus replication or BKPyVAN.

Study	Design/population	BK viral load at ECP initiation	ECP regimen/follow-up	Rejection and main outcomes
Xipell 2022, J Clin Apher	Single-center case series; index case = BKPyVAN after prior ABMR	~7.0 log10 copies/ml	11 sessions over 27 weeks (2/week × 2 week → weekly × 2 week → q2wk); follow-up 6 months	VL ↓ ~90% at 14 days and 99.9% at 6 months; graft function stable on ECP, worsened after stopping; low-titer de novo anti-HLA-A*01 DSA (MFI 1790); no ECP-related complications
Von Tokarski 2024, BMC Nephrol	Case report; kidney Tx in prior double-lung recipient	6.9 log10 (POD15) → 8.0 log10 (POD28)	ECP from POD44 to support IS minimization; follow-up 1 year	VL ↓ to 3.4 log10 at month 4, negative at months 9 and 11; no lung/kidney rejection; stable function
Escudero-Saiz 2025, Clin Kidney J	Single-center retrospective series; 13 kidney Tx, refractory biopsy-proven BKPyVAN	Median 6.56 log10 copies/ml (3.66 × 10⁶; IQR 0.85–7.71 × 10⁶)	Rescue ECP; median 11 sessions (IQR 10–20) over 19 weeks (IQR 11–35); median follow-up 12 months (IQR 6–18)	VL ↓ 55.3% at 3 months, 99.9% at 18 months; 1 de novo chronic-active ABMR (no major graft impact); 2 graft losses (BKPyVAN); no ECP-related AEs
Gregorini 2025, Front Nephrol	Case report; highly sensitized second kidney Tx	3.71 log10 at ECP start (BKPyVAN peak 5.21 log10/162 200 copies/ml)	2 procedures/week × 3 week → fortnightly × 2–3 → monthly, later q2mo; follow-up 2023–2025	No acute rejection; sustained viral control; stable renal function; anti-DR53 DSA declined

Abbreviations: ABMR, antibody-mediated rejection; AE, adverse event; BKPyVAN, BK polyomavirus-associated nephropathy; DSA, donor-specific antibody; ECP, extracorporeal photopheresis; IQR, interquartile range; IS, immunosuppression; MFI, mean fluorescence intensity; POD, postoperative day; Tx, transplantation; and VL, viral load.

## PRACTICAL CONSIDERATIONS: TREATMENT SCHEDULES AND PATIENT SELECTION

Notwithstanding these limitations, practical guidance on treatment schedules and patient selection may be useful for clinicians considering ECP in this context. The optimal treatment schedule for ECP remains to be standardized. Across transplant settings, protocols typically consist of two consecutive sessions per cycle, administered initially on a weekly or biweekly basis, followed by monthly maintenance phases. However, the schedule also varies depending on the type of apheresis device’s technology used to perform the ECP. When the connected technology is used, 1.5 l of blood is processed in each session that is repeated twice a week. In total, about 28 sessions are performed. In the case of the disconnected technology, one total blood volume is treated in each session being considered one therapeutic cycle; the patients underwent ECP twice weekly during the initial 2 weeks, followed by weekly sessions until the first month was completed. Thereafter, treatment continued every 2 weeks until a minimum of 14 sessions were reached [33, 64].

In kidney transplantation, reported regimens generally include ~10–15 sessions in the case of the disconnected technology or 20–30 sessions if the connected technology is used during the first months of treatment [24]. However, treatment duration is usually individualized according to viral load kinetics, immunological response, and patient tolerance. ECP may be particularly relevant in patients with persistent viremia despite immunosuppression reduction or in those at high immunological risk in whom further reduction is not feasible.

## SAFETY AND FEASIBILITY OF ECP

ECP is generally considered a well-tolerated procedure, and the use of the disconnected technology reduces to a half the number of procedures and the burden that this represents for the apheresis centers. In the World Apheresis Association registry, adverse events occurred in 2.1% of 13 871 ECP procedures, severe events were rare, 0.04%, and no procedure-related deaths were reported [65, 66].

Expected complications include vascular-access problems, citrate-related symptoms, transient hypotension, nausea, fever or chills, local bleeding or thrombophlebitis, and, with prolonged treatment, possible decreases in blood counts [35, 67]. However, the use of ultrasonography for cannulation of peripheral veins reduces the need of central lines and the complications associated to its use [68]. For instance, in our institution, 98% of the ECP procedures performed in 2023 were done using peripheral veins [33].

In solid-organ transplant recipients, adverse events were reported in 1.7% of procedures, with no severe events in that subgroup. In the previous 13-patient series, no procedure-related adverse events or intercurrent infections were observed; however, this should be interpreted cautiously given the small retrospective design and treatment in an experienced center [24].

## DISCUSSION AND FUTURE PERSPECTIVES

Current management of BKPyVAN relies on systematic monitoring of viremia and stepwise reduction of immunosuppression. However, the absence of effective antiviral therapies remains a major limitation, particularly in patients at high immunological risk, in whom immunosuppression minimization may increase the likelihood of rejection. This therapeutic dilemma has driven interest in immunomodulatory strategies capable of restoring antiviral immunity without compromising graft tolerance.

Clinical experience with ECP in BKPyV infection remains limited to uncontrolled case series, with indirect support from its association with reduced viral burden—particularly CMV—in other transplant settings. In kidney transplantation, available data suggest viral load reductions and immunosuppression adjustment without apparent increase in rejection risk, though these observations are hypothesis-generating rather than conclusive.

These mechanistic effects provide a rational basis for the clinical observations described above, including induction of tolerogenic dendritic cells, expansion of regulatory T cells, modulation of cytokine profiles, and potential antifibrotic effects. Rather than functioning as a conventional immunosuppressive therapy, it has been hypothesized that ECP might promote immune rebalancing and selectively enhance antiviral immunity without broadly compromising alloimmune tolerance—a proposed “decoupling” that, if confirmed, could enable more controlled immunosuppression reduction while preserving graft integrity. However, whether such differential modulation of antiviral versus alloimmune responses actually occurs with ECP has not been directly demonstrated; no available study in BKPyV infection has assessed virus-specific T-cell responses before or after ECP, and this represents a fundamental mechanistic gap that prospective studies should address.

Within the current therapeutic landscape, immunosuppression reduction remains the cornerstone of BKPyV management, and conventional adjunctive agents—IVIG, leflunomide, and cidofovir—are used empirically alongside it, albeit without consistent efficacy in controlled settings. ECP occupies a niche as an adjunctive strategy in patients with persistent viremia or BKPyVAN refractory to these measures, particularly those at high immunological risk in whom further immunosuppression reduction is not feasible. Intervention at early stages, before significant fibrosis has developed, is likely to be most relevant, as established chronic graft injury limits the potential benefit of any immunological intervention, including virus-specific T-cell therapy. Virus-specific T-cell therapy is the most mechanistically targeted strategy in development, directly restoring BKPyV-specific immunity, but remains investigational and requires specialized infrastructure. ECP, in contrast, has an established safety profile in transplantation, acts through broader immunomodulatory mechanisms that may simultaneously reduce rejection risk, and is accessible in centers with existing apheresis programs.

Several limitations temper these conclusions. No randomized controlled trials have evaluated ECP specifically in BKPyV infection; available data derive exclusively from case reports and small, uncontrolled case series, two of which originate from the same center, raising the possibility of center-effect bias. Publication bias cannot be excluded, as favorable outcomes are inherently more likely to be reported than treatment failures or complications. Survivor bias is also a concern, since patients who tolerate prolonged ECP regimens and remain available for follow-up may differ systematically from those who discontinue treatment or experience early graft loss. Finally, in nearly all reported cases, ECP was administered alongside immunosuppression reduction, precluding attribution of the observed virological responses to ECP itself.

Also, although available data support a favorable procedural safety profile, ECP should be regarded as a specialized adjunctive strategy whose feasibility depends on patient selection, vascular access, center expertise, logistics, and cost.

Taken together, current evidence suggests that ECP may represent a valuable adjunctive strategy in selected patients with BKPyV replication or BKPyVAN, particularly in high-risk clinical scenarios. This review provides a comprehensive and up-to-date synthesis of the available evidence supporting this approach.

However, the evidence base remains limited, and prospective studies and controlled clinical trials are needed to better define the role of ECP, including optimal timing of initiation, patient selection, and treatment schedules.

## Data Availability

No new data were generated or analyzed in support of this research.
